# Synthesis of Quercetin-Loaded Silver Nanoparticles and Assessing Their Anti-Bacterial Potential

**DOI:** 10.3390/mi14122154

**Published:** 2023-11-25

**Authors:** Ritu Sharma, Parakh Basist, Abdulsalam Alhalmi, Rahmuddin Khan, Omar M. Noman, Ahmad Alahdab

**Affiliations:** 1 School of Medical and Allied Sciences, K.R. Mangalam University, Gurugram 122103, India; ritu01sharma@gmail.com; 2 Department of Pharmacology, School of Pharmaceutical Education and Research, Jamia Hamdard, New Delhi 110062, India; 3 Department of Pharmaceutics, School of Pharmaceutical Education and Research, Jamia Hamdard, New Delhi 110062, India; asalamahmed5@gmail.com (A.A.); rkm.hamdard@gmail.com (R.K.); 4 Department of Pharmacognosy, College of Pharmacy, King Saud University, P.O. Box 2457, Riyadh 11451, Saudi Arabia; 5 Institute of Pharmacy, Clinical Pharmacy, University of Greifswald, Friedrich-Ludwig-Jahn-Str. 17, 17489 Greifswald, Germany

**Keywords:** quercetin, silver nanoparticles, antibacterial

## Abstract

The study delves into the multifaceted potential of quercetin (Qu), a phytoconstituent found in various fruits, vegetables, and medicinal plants, in combination with silver nanoparticles (AgNPs). The research explores the synthesis and characterization of AgNPs loaded with Qu and investigates their pharmaceutical applications, particularly focusing on antibacterial properties. The study meticulously evaluates Qu’s identity, and physicochemical properties, reaffirming its suitability for pharmaceutical use. The development of Qu-loaded AgNPs demonstrates their high drug entrapment efficiency, ideal particle characteristics, and controlled drug release kinetics, suggesting enhanced therapeutic efficacy and reduced side effects. Furthermore, the research examines the antibacterial activity of Qu in different solvents, revealing distinct outcomes. Qu, both in methanol and water formulations, exhibits antibacterial activity against *Escherichia coli*, with the methanol formulation displaying a slightly stronger efficacy. In conclusion, this study successfully synthesizes AgNPs loaded with Qu and highlights their potential as a potent antibacterial formulation. The findings underscore the influence of solvent choice on Qu’s antibacterial properties and pave the way for further research and development in drug delivery systems and antimicrobial agents. This innovative approach holds promise for addressing microbial resistance and advancing pharmaceutical formulations for improved therapeutic outcomes.

## 1. Introduction

Quercetin (Qu) is considered as the most significant phytoconstituent which is an organizing component for many other flavonoids. It is a particular kind of flavonoid found in several vegetables and fruits, including onions, berries, apples, tea, and brassicas, as well as well as many seeds, flowers, nuts, barks, leaves, and medicinal plants such as *Ginkgo biloba*, *Piper cubeba*, *Solanum trilobatum*, *Withania somnifera*, etc [1]. In the United States, an expected daily dietary intake of Qu is 25 mg which is a vital component in multiple nutraceuticals and food supplements [2].

The IUPAC name of Qu is 3,5,7,3′,4′-pentahydroxy flavone, suggesting it is a component of the flavonoids family. It has three rings with five hydroxyl groups, and two aromatic rings A and B connected by a heterocyclic ring C that contains oxygen. The B ring contains multiple hydroxyl groups. The utilization of Qu glycosides in the small intestine is typically low.

Qu antibacterial activity arises from its interference with bacterial DNA gyrase, cell membrane disruption, antioxidant properties, and interference with quorum sensing. However, its use faces challenges, including a limited bioavailability and poor aqueous solubility. These limitations affect its effectiveness against systemic infections and in aqueous environments. Additionally, bacterial efflux pumps can reduce its intracellular concentration, and its antibacterial activity may vary among different bacterial strains. Researchers are working on strategies to improve its solubility, bioavailability, and overall antibacterial efficacy for potential therapeutic applications against pathogenic bacteria.

Qu has stronger antioxidant properties compared to Trolox, ascorbyl, and rutin because it has more and strategically positioned free hydroxyl groups. When flavonoid glycosides are absorbed in the small intestine or processed by bacteria in the colon, they transform into a form of Qu called quercetinaglycones. This transformed Qu can also change into sulphated or glucuronidated forms. Qu has the right structure for activities like binding to ions and scavenging free radicals, which makes it well-known for its antioxidant capabilities [3].

Similar to this, the anti-cancer potential results from the removal of free radicals, the suppression of an enzyme that activates carcinogens, the modification of signal transduction pathways, interactions between estrogen receptors, and other protein interactions. Additionally, anticancer effects were investigated, which included lowering MMP-2 and MMP-9 synthesis in prostate cancer cells, activating apoptosis and fatty acid synthase, and reducing cell proliferation [4].

Due of the wide range of applications for nanotechnology, including drug administration, cell labelling, cancer therapy, anti-microbial medicines, and diagnostics, progress in this field has remained consistent on a global scale [5]. Nanoparticles have distinctive features that depend on their morphology, shape, and size, which permit them to interact with plants and animals, as well as microorganisms [6].

AgNPs excel in antibacterial applications due to their potent, broad-spectrum activity and reduced resistance development. Their synergy with antibiotics further enhances their efficacy. Green synthesis methods, incorporating natural sources and eco-friendly processes, are convenient for AgNPs’ production. These methods offer environmental benefits, such as reduced energy consumption and lower costs. They result in biocompatible nanomaterials with controlled properties, making them a sustainable and effective choice for antibacterial applications in areas like biomedicine and environmental remediation, while also aligning with eco-friendly and cost-effective practices [7].

Against several bacteria, silver nanoparticles (AgNPs) have been reported to exhibit excellent bactericidal capacities. AgNPs are beneficial to the environment due to their application in catalysis, electronics, and medicines, as well as regulating microorganisms’ growth in biological systems. AgNPs can be created using a wide range of approaches, comprising chemical, physical, and biological ones. The AgNPs have a constrained size distribution, giving physical techniques an additional advantage over chemical ones. On the other hand, the principal limitations are significant energy consumption [8].

The production of an extensive range of synthetic materials, including carbon nanotubes, polymer-coated silver, metal oxides, dendrimers, nanofilms, and nanofibers, are the main usage of NPs. The use of metal elements at nanometric dimensions for antibacterial, optical, catalytic, electrical, and sensing applications is common. These metals comprise Ag, Au, Fe, Cu, Pt, Pd, Ni, and Co. A great deal of interest has been expressed in the biosynthesis of NPs, especially AgNPs from plant extracts or organic sources due to the numerous advantages and wide variety of bioactive reducing metabolites [9,10].

This research offers an innovative approach to creating Qu-loaded AgNPs with unique elements. It explores Qu derivatives’ impact on nanoparticle stability and antibacterial properties, goes further by developing a biocompatible coating, delves into molecular-level insights, and addresses environmental and safety considerations. These aspects distinguish it from prior studies.

Due to the large surface area to volume ratio and antibacterial characteristics, AgNPs are thought to be the most promising due to the emergence of resistant strains and growing microbial resistance to metal ions, antibiotics, and other chemicals. Thus, the current research project aims to generate AgNPs that amplify Qu antibacterial activity while minimizing adverse effects and improving medication therapeutic efficiency. Therefore, AgNPs are selected as the drug’s distribution vector.

## 2. Materials and Methods

### 2.1. Material

The Qu (97% pure) and silver nitrate were purchased from Afflatus Pharmaceuticals Pvt. Ltd., Haryana, India, and Thermo Fisher Scientific Pvt. Ltd., Mumbai, India. Other chemicals including tri sodium citrate was procured from Nice chemicals Pvt. Ltd., Mumbai, India. Methanol was obtained from LobaChemiePvt. Ltd., Mumbai, India, and other solvents and chemicals employed were of analytical grade.

### 2.2. Organoleptic Characteristics

For the sake of identifying the medication, visual observations were used to conduct organoleptic research on factors like general appearance, such as color, nature, and odor. For color analysis, a small amount of the drug was taken on butter paper and observed in an area with adequate illumination, whereas for observing the odor, a small quantity of the drug was smelled in order to obtain the fragrance [11].

### 2.3. Detection of Melting Point

The USP method was adopted to calculate the melting point. An enclosed capillary tube contained a small amount of the medication. The melting point device was filled with the tube. The apparatus’s temperature was steadily raised, and it was noticed at what point the medication began to melt and at what point the full amount of the drug melted [12].

### 2.4. Dual Scanning Calorimetry (DSC)

For DSC tests the drug, the DSC TA 60 Shimadzu thermal analyzer was utilized. To calibrate the instrument, high purity indium metal was used as a reference. The scans were conducted under a nitrogen atmosphere at a heating rate of 10 °C per minute [13].

### 2.5. FTIR Study of Qu Nanoparticles

FTIR spectroscopy (Perkin Elmer, Rodgau, Germany) was employed to ascertain the contribution of the functional groups of Qu-AgNPs. This analysis was carried out at a resolution of 4 cm^−1^, utilizing a KBr disk.

### 2.6. Spectroscopy Used for UV–Visible Analysis

#### 2.6.1. Identifying the Wavelengths of Qu

For identifying Qu wavelengths, a UV–Vis spectrophotometer with a 1 cm quartz cuvette is employed. The experimental conditions entail dissolving Qu in an appropriate solvent, like water and ethanol, with a standard concentration. The wavelength range spans 200–800 nm, baseline correction is applied, and measurements are made at room temperature. A common standard scan speed for UV–Vis spectrophotometers is 200 nm/min.

#### 2.6.2. Establishing the Ethanol Standard Curve

To establish a standard calibration curve for quercetin in ethanol, begin by preparing a quercetin stock solution of known concentration, i.e., by dissolving 10 mg of quercetin in 10 mL of ethanol to achieve a stock solution with a concentration of 1 mg/mL. From this stock solution, generate a range of standard solutions with different concentrations, such as 0.1 mg/mL, 0.5 mg/mL, 1.0 mg/mL, 2.0 mg/mL, 5.0 mg/mL, and 10.0 mg/mL, by diluting the stock solution accordingly. Measure the absorbance of each standard solution at a specific wavelength 420 nm using a UV–Vis spectrophotometer, ensuring a uniform cuvette type, path length, and spectrophotometer settings for all measurements. Record the absorbance values corresponding to each known concentration in a data table. This calibration equation can subsequently be utilized to determine the concentration of quercetin in unknown samples based on their measured absorbance values, facilitating quantitative analysis [14].

### 2.7. AgNP Synthesis

TSC is employed in the production of AgNP as a reducing agent. The synthesis of AgNP involves the use of silver nitrate and TSC as initial components. Chemical reduction was used to create the silver colloid. In distilled water, all solutions of the substances that react are formed. A typical experiment involved boiling 50 mL of 0.001 M AgNO_3_. Drop by drop, 5 mL of 1% trisodium citrate was incorporated to this solution. Solutions were heated and aggressively stirred throughout the procedure until an apparent color shift (pale yellow) occurred. It was then taken out of the heater and swirled until it reached room temperature. AgNPs were combined with 2 g of Qu that had been dissolved in methanol [15].

### 2.8. Nanoparticles Evaluation

#### 2.8.1. Effective Drug Trapping

The composition of the nanoparticle suspension was assessed through a series of measurements. Initially, 5 mL of this formulation was diluted with distilled water to reach a total volume of 8 mL. The separation of components was achieved using a high-speed centrifuge (Remi Pvt Ltd., Maharashtra, India). The centrifugation process was carried out for 45 min at an impressive speed of 30,000 rpm, with the cooling system active to maintain optimal conditions. After centrifugation, the resulting mixture underwent phase separation, yielding a supernatant and sediment. The volumes of both fractions were carefully calculated. Subsequently, the sediment was lysed utilizing *n-*propanol and then passed through a 0.4 µm nylon disk filter to further purify the sample. The final step involved the quantification of Qu content in both the supernatant and sediment. This was accomplished by employing a UV spectrophotometer, with measurements taken at a specific wavelength of 420 nm, allowing for the precise determination of Qu concentrations in each fraction [16]. The following equation was applied to calculate the percentage of drug entrapment:

$$\mathrm{Percentage}\;\mathrm{entrapment}\;\mathrm{efficiency}=\frac{amount\;of\;drug\;recovered}{total\;ampount\;of\;drug}\times 100$$

#### 2.8.2. Shape and Surface Morphology of Nanoparticles

TEM (Philips Technai electron microscope, Eindhoven, The Netherlands) was employed to visualize the nanoparticles. A drop of nanoparticle solution was allowed to evaporate on a microscopic carbon grid, allowing the excess to be removed through filter paper. The sample was then subjected to a drop of a 1% phosphor tungstic acid (PTA) aqueous solution for 5 min. Before examining the vesicles under a TEM running at a 200 KV acceleration voltage, the surplus solution was drained off of the sample, which was then allowed to dry at room temperature [17].

#### 2.8.3. Particle Size Analysis

The size of nanoparticles was assessed using the dynamic light scattering method (DLS) with the aid of computerized inspection equipment, specifically the Malvern Zetasizer Nano-ZS, and DTS nano software (version 6.34). Utilizing distilled water to dilute the nanoparticle solution, zetasizer cuvettes were employed to measure the size of the particles. Next, measurements were made at a temperature of 25 °C. At each temperature, the sample was equilibrated for at least 3 min prior to the measurement. The DLS measurements were conducted during alternating increasing and decreasing temperature cycles. The Z-average value obtained from DLS corresponds to the nanoparticles under consideration’s average hydrodynamic diameter. In order to better understand vesicle size and size distribution, data were gathered [18].

#### 2.8.4. Measuring Zeta Potential

Zeta potential (Zetasizer-1000HS (Malven Instruments, Malvern, UK) is a characteristic of a substance determined by the overall surface charge of nanoparticles. In this study, an electrolyte suspension of NaCl (2 × 10^−2^ M NaCl), was prepared by diluting it with 50 mL double-distilled water extracted from liquid nanoparticle samples of 5 mL. The pH of the suspension was then adjusted to the desired level. After 30 min, the samples were collected, and following agitation, the equilibrium pH was measured to assess the zeta potential of the metallic particles. The surface potential of the AgNP was computed using the zeta potential. An average of three measurements was recorded for each scenario. The stability of NPs was evaluated based on the zeta potential values, ranging from higher than +30 mV to lower than −30 mV [19].

#### 2.8.5. Diffraction of X-rays

The interference generated by the scattering of X-rays by a crystal’s atoms allows the diffraction pattern to provide information about the crystal’s structure or the nature of a crystalline material. A glass slide was coated with 1 mL of the AgNPs solution, which was then dried in an oven with a temperature of 40 °C. To produce a thin film, the technique had to be carried out three to four times. Cu K radiation, 1 = 1.54056; 2 = 1.54439, was used to produce the spectra in a Phillips Xpert Pro Diffractometer operating at 40 kV and 30 mA. From the angles 35.01° to 79.99°, the diffracted intensities were measured [20].

#### 2.8.6. Studies on Drug Release In Vitro

Multiple kinetic models, such as the Higuchi model, zero-order model, first-order model, log cumulative% model, and Korsmeyer–Peppas model, were utilized to graphically represent data from the in vitro drug release experiments and analyze the release kinetics of the nanoparticles. Various release kinetics were studied by employing different plots, including the zero-order plot, first-order plot, Higuchi plot, and Korsmeyer–Peppas plot. The high correlation coefficient, which approached 1, confirmed the suitability of the model that provided the best fit for the data [21].

#### 2.8.7. Analysis of Antibacterial Activity

To evaluate the antibacterial activity against *Escherichia coli*, the agar well diffusion method was employed. This method involves using the disc diffusion technique to measure bacterial inhibition. Test substances are placed within a disc, and the diameter of the zone of inhibition is subsequently measured. In the context of this study, the antibacterial activity of aqueous and solvent extracts was assessed using the agar well diffusion method. Initially, an inoculum containing 106 cfu/mL of the *E. coli* bacterial culture under examination was evenly spread on agar plates using a sterile swab soaked in the bacterial suspension. Subsequently, 8 mm-diameter wells were created in the agar medium and filled with 100 μL of Qu-AgNPs dissolved in methanol. These plates were allowed to sit for 2 h at room temperature to facilitate diffusion. Following this, the plates were incubated for 24 h at 37 °C in an upright position, and the resulting data were presented as a mean [22,23,24].

## 3. Results

### 3.1. Organoleptic Characteristics

To verify the authenticity of the medication, we compared the observed characteristics with the established standards outlined in the pharmacopeia. It was determined that the observed attributes met the specified requirements [25]. The color of Qu was observed to be yellow crystalline, whereas the odor was found to be pungent. The observed results were found to be comparable with that of the pharmacopeia. The drug’s sensory characteristic is a crucial procedure for confirming both the drug’s identity and its overall quality.

### 3.2. Melting Temperature

The melting point of Qu was determined to be 316 °C. Three separate measurements of the melting point were conducted, and the average value was documented [26]. The consistency in the melting point across these multiple measurements serves as an indicator of the drug’s purity and stability.

### 3.3. Dual Scanning Calorimetry (DSC)

**Figure 1:**
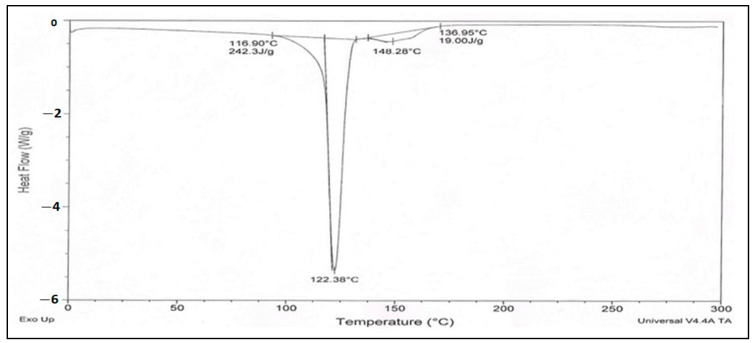
DSC thermogram of quercetin.

In the DSC thermograms, a distinct endothermic peak was evident, aligning with the melting point of Qu at 316 °C [27]. Figure 1 illustrates the DSC thermogram for Qu, reinforcing the precision of the melting temperature determination.

### 3.4. Measurement of pH

The pH of Qu was determined to be 2.2 which is comparable with the previous results [28]. This information is vital for assessing the drug’s compatibility with physiological conditions and potential effects on gastrointestinal absorption.

### 3.5. FTIR Study of Qu Nanoparticles

**Figure 2:**
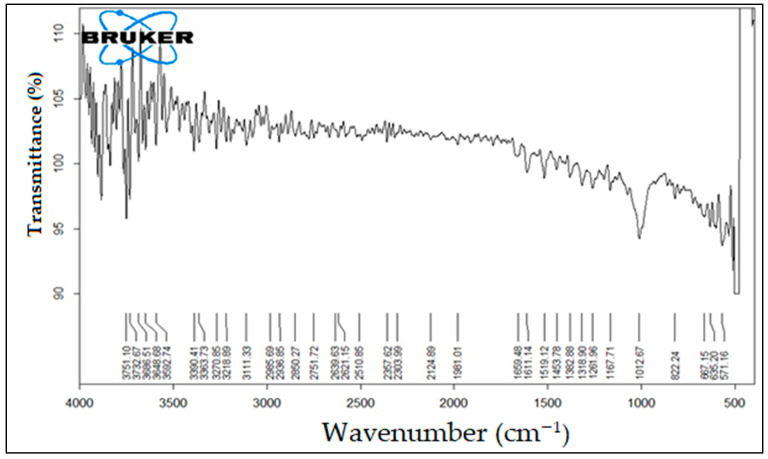
FTIR spectrum of quercetin.

To characterize the medication, an analysis using FTIR spectroscopy was conducted. These unique peaks play a crucial role in identifying the medication. In the context of medication compatibility testing, FTIR analysis of Qu-AgNPs was performed, as depicted in Figure 2. It was observed that all spectral groups exhibited consistent values, affirming the authenticity of the drug sample and confirming the absence of any discernible impurities [29].

FTIR spectroscopy was used to analyze the chemical composition of the reactants involved in Qu-AgNPs’ synthesis. The spectrum displayed a broad peak at 3732.67 cm^−1^, indicating O-H stretching vibrations associated with the hydroxyl groups in quercetin (Qu). Other peaks were observed at 1519.12 cm^−1^ and 1659.48 cm^−1^ which correspond to the stretching and bending vibrations of these aromatic rings. Furthermore, a 1453.78 cm^−1^ band indicated C=C stretching in the aromatic compounds, while a 1012.67 cm^−1^ band represented the stretching vibrations of the C-O bonds in quercetin. These findings provide evidence of quercetin’s role in both reducing and stabilizing the synthesis of Qu-AgNPs.

### 3.6. Spectrophotometry (Standard Graph Development)

**Figure 3:**
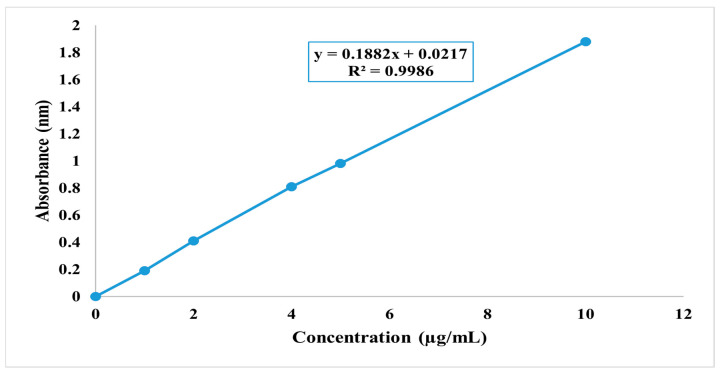
Standard calibration curve of quercetinin ethanol at 420 nm.

The development of a standard graph for Qu’s absorption at 363 nm in ethanol allowed for quantitative analysis. This method is valuable for accurately determining the drug concentration in solutions. A Qu standard solution was prepared by combining 10 mg of Qu with 10 mL of ethanol, resulting in the creation of a stock solution with a concentration of 10 µg/mL. The highest absorption point for Qu was determined to be at 420 nm following the scanning of dilutions ranging from 0.1 to 10 mg/mL across the 200–600 nm range, as depicted in Figure 3. Subsequently, a calibration curve in ethanol was constructed using solutions from the above-mentioned range, with concentrations set at 1, 2, 4, 5, and 10 µg/mL [30].

### 3.7. Nanoparticle Evaluation

#### 3.7.1. Efficacy of Drug Entrapment

The high entrapment efficiency of 85.4% demonstrates the effectiveness of the formulation method, surpassing reported efficiencies in nanoparticle drug delivery systems [31].

#### 3.7.2. Transmission Electron Microscopy (TEM)

**Figure 4:**
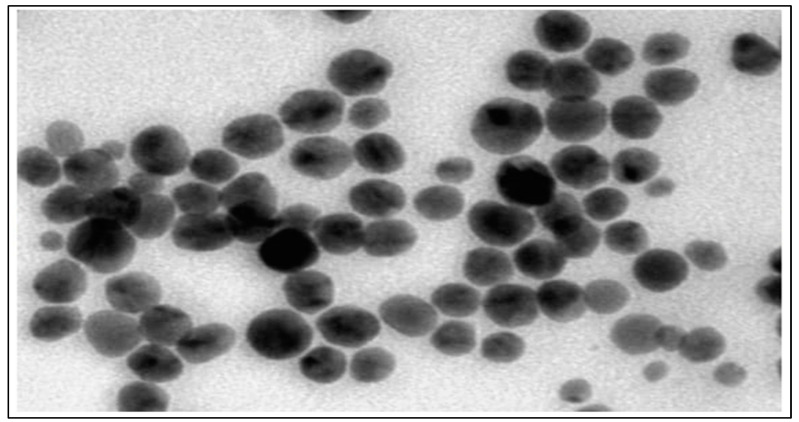
TEM image of silver nanoparticles.

To generate an image of nanoparticles with a scale bar set at 200 nm and a magnification of 13.0 × 4000, TEM was chosen as the preferred method. Subsequently, TEM analysis was conducted on the selected formulation, revealing the presence of spherical, single-layer vesicles characterized by a smooth surface [32]. This structure is deemed advantageous for improving drug solubility and enhancing its bioavailability, as depicted in Figure 4.

#### 3.7.3. Zeta Potential Calculation

**Figure 5:**
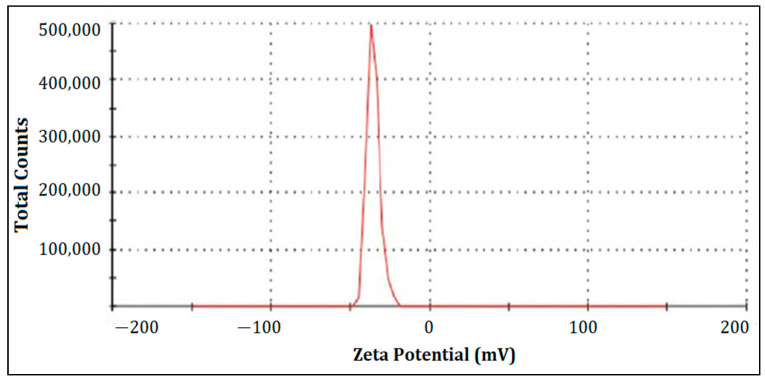
Zeta potential of nanoparticles.

The zeta potential is −15.1 + 3.60 mV indicates a good nanoparticle stability, crucial for preventing aggregation and ensuring a longer shelf life as shown in Figure 5.

#### 3.7.4. Measurement of Particle Size

**Figure 6:**
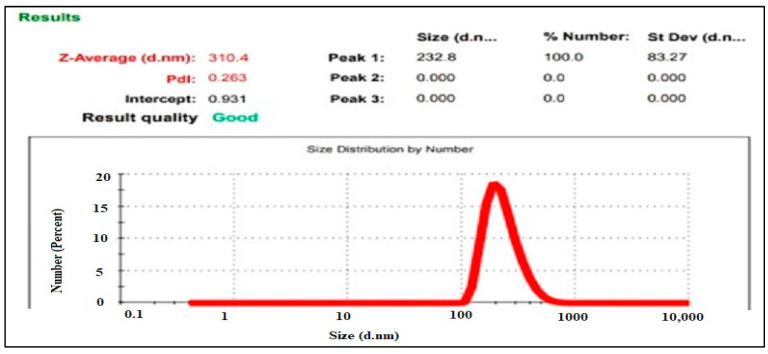
Particle size of the prepared nanoparticles.

The Z-average particle size of 310.4 nm as shown in Figure 6 falls within the typical range for drug delivery nanoparticles, making them suitable for potential therapeutic applications.

#### 3.7.5. X-ray Diffraction (XRD) Pattern

**Figure 7:**
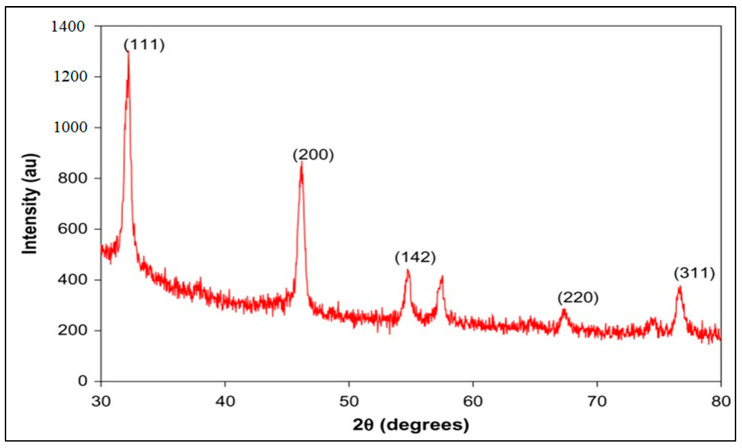
X-ray diffraction of nanoparticles.

AgNPs display a crystalline structure, according to XRD. The crystalline structure observed in X-ray diffraction (XRD) analysis suggests alterations in Qu’s physicochemical properties, potentially improving its dissolution rate and bioavailability. The X-ray diffraction is shown in Figure 7.

#### 3.7.6. Examination of In Vitro Release

The in vitro release kinetics, best fitting the first-order model, suggested controlled and sustained drug release, enhancing therapeutic efficacy and reducing potential side effects. These findings collectively support the potential utility of Qu nanoparticles in pharmaceutical formulations, especially for poorly soluble drugs like Qu, with implications for improved drug delivery and therapeutic outcomes [33].

The in vitro drug release kinetics of Qu from the pH 7.4 formulation in PBS were investigated using various kinetic models, and the results are presented graphically represented in zero-order, first-order, Higuchi, and Korsmeyer–Peppas plots.

**Figure 8:**
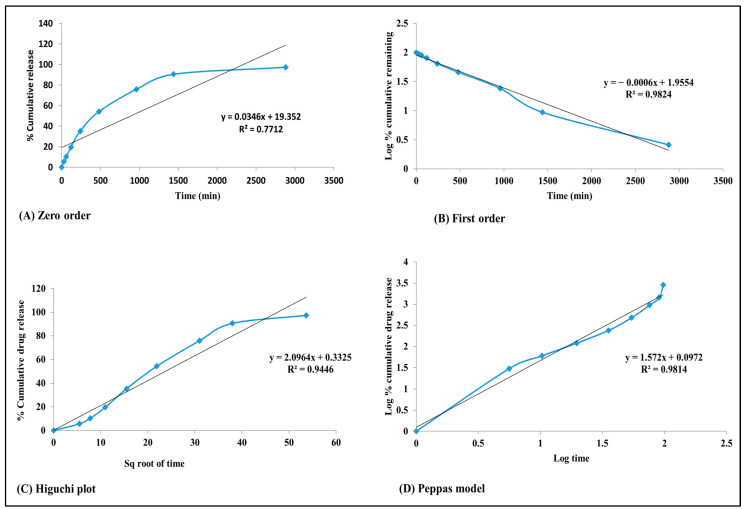
The in vitro drug release kinetics of Qu from the pH 7.4 formulation in PBS, (**A**) zero-order kinetic, (**B**) first-order kinetic, (**C**) Higuchi model release, and (**D**) Peppas model release.

In the zero-order plot, cumulative drug release was plotted against time. This model assumes a constant release rate over time. The R^2^ value of 0.771 suggests that the zero-order model is not the best fit for Qu release, indicating that the drug release from this formulation is not a constant-rate process as shown in Figure 8A.

The first-order plot shows the remaining cumulative log of the medication over time, which is characteristic of a first-order kinetic release profile. The high R^2^ value of 0.982 indicates an excellent fit for the kinetic model of first order. This suggests that the release of Qu from the formulation follows a first-order rate process, where a constant fraction of the drug is released per unit of time. This information is valuable for designing controlled-release dosage forms where a consistent release rate is desired to maintain therapeutic drug levels over time as shown in Figure 8B.

The Higuchi model entails graphing the cumulative drug release rate against the square root of time. This model is typically applied in situations where drug release is primarily governed by diffusion within a matrix as shown in Figure 8C. The relatively high R^2^ value of 0.944 indicates that the Higuchi model fits the data quite well, suggesting that diffusion might be a significant factor in the release of Qu [34].

The Korsmeyer–Peppas model involves plotting the log percent cumulative release of medication against log time. This model is often used to characterize drug release from polymeric systems and can help to identify the release mechanism. The R^2^ value of 0.981 indicates a strong fit for the Korsmeyer–Peppas model, suggesting that the release of Qu may involve a combination of diffusion and polymer erosion, which is typical for many controlled-release systems as shown in Figure 8D [31].

Thus, the in vitro release data for Qu from the pH 7.4 formulation in PBS were analyzed using multiple kinetic models [35]. The first-order kinetic model was found to best describe the drug release process, indicating a consistent fraction of drug release per unit time. This finding is valuable for the development of pharmaceutical dosage forms with controlled release profiles, ensuring optimal therapeutic outcomes. Additionally, the strong fits observed in the Higuchi and Korsmeyer–Peppas models suggest that diffusion and polymer erosion may also contribute to the release mechanism, providing insights into the underlying release processes of Qu from this formulation.

### 3.8. Antibacterial Activity

The assessment of antibacterial activity against *E. coli* using the disc diffusion method provides valuable insights into the potential therapeutic applications of Qu-AgNPs in different solvents. The results indicate distinct outcomes for two different formulations: Sample 1, containing water and Qu-AgNPs, and Sample 2, where Qu-AgNPs are in methanol [32].

**Figure 9:**
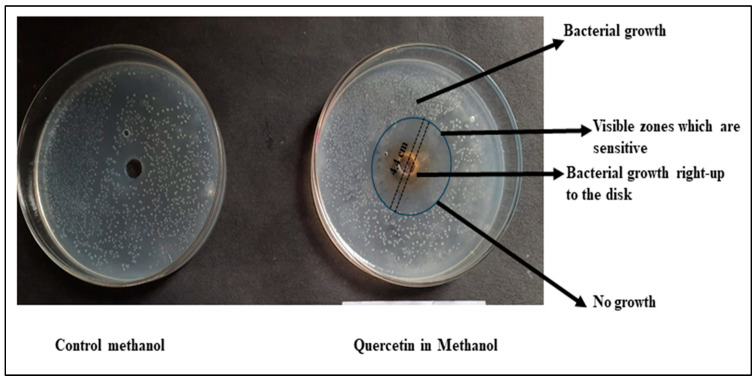
Qu-AgNPs–methanol-based antibacterial effect at concentration 10 mg/mL.

In Sample 2, the observed delicate antibacterial activity with a zone of inhibition measuring 4.4 cm suggests that the methanol and Qu-AgNPs formulation has a relatively stronger antibacterial effect against *E. coli*. This result aligns with the existing literature, which highlights Qu-AgNPs’ potential as an antimicrobial agent. The presence of methanol in this sample might enhance Qu’s solubility and bioavailability, potentially contributing to its increased antibacterial activity [36]. Further investigation is warranted to elucidate the underlying mechanism of action and to assess the potential of this formulation in the development of antimicrobial agents as shown in Figure 9.

**Figure 10:**
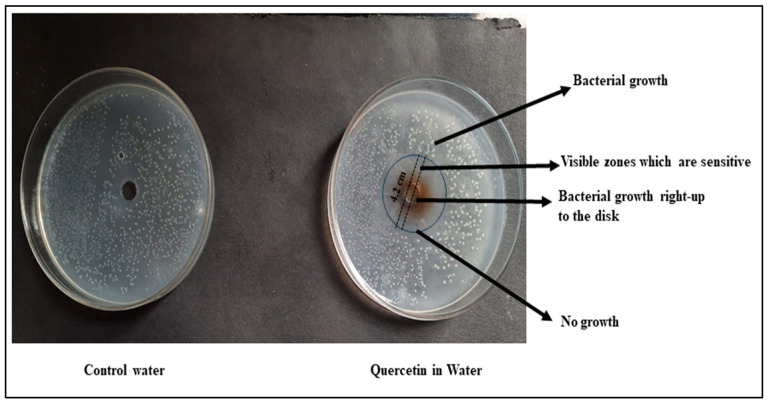
Qu-AgNPs–water-based antibacterial effect at concentration 10 mg/mL.

In contrast, i.e., in Sample 1, the zone of inhibition of 4.2 cm observed for Qu in water, while slightly smaller than that of Sample 2, still indicates fragile antibacterial activity. This result underscores the inherent antimicrobial properties of Qu, even when solubilized in water. Qu’s ability to inhibit bacterial growth in an aqueous medium is of particular interest in the context of developing natural antimicrobial agents for various applications, including food preservation and pharmaceuticals as shown in Figure 10.

**Figure 11:**
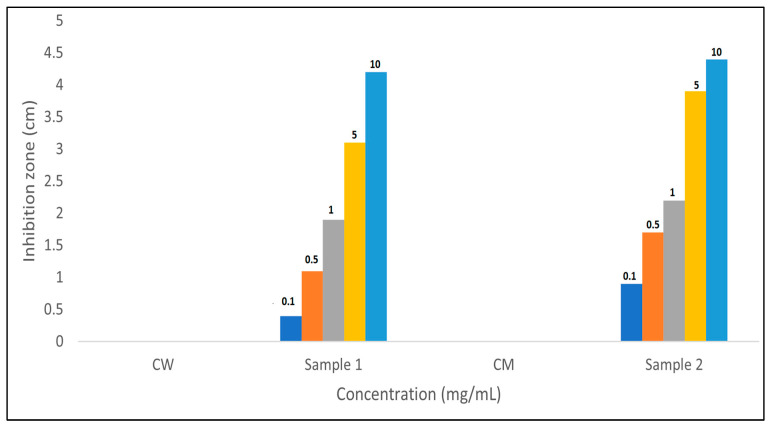
The bar graph summarizes the results of antibacterial assay, displaying the zone of inhibition for each sample in cm, including Sample 1 (Qu-AgNPs in water), Sample 2 (Qu-AgNPs in methanol), CW (control water), and CM (control methanol) at different concentration 0.1, 0.5, 1, 5, and 10 mg/mL.

Thus, the disc diffusion method revealed that both methanol and water formulations of Qu exhibit antibacterial activity against *E. coli*. Sample 1, with methanol, displayed a slightly larger zone of inhibition, suggesting enhanced antibacterial efficacy compared to Sample 2 in water (Figure 11). These findings support the potential use of Qu as an antimicrobial agent and highlight the influence of solvent choice on its antibacterial properties. Further investigations, including detailed antimicrobial mechanism studies, are warranted to explore the full therapeutic potential of Qu as an antibacterial agent in various formulations.

## 4. Discussion

The results presented in this study encompass a comprehensive evaluation of Qu, a naturally occurring flavonoid with potential pharmaceutical applications. The study employed various analytical techniques to assess the drug’s identity, quality, solubility, physicochemical properties, and potential therapeutic applications in different formulations. These findings shed light on the multifaceted aspects of Qu and its suitability for pharmaceutical development.

The organoleptic characteristics of the drug, including its appearance, taste, and odor, were evaluated and found to comply with pharmacopeial standards, ensuring its identity and quality [37]. The determination of Qu’s melting temperature at 316 °C through multiple measurements corroborated its purity and stability, further reinforcing its suitability for pharmaceutical use [38]. The presence of a prominent endothermic peak in the DSC thermogram at the same temperature as the melting point provided additional evidence of the accuracy of the melting temperature determination. Solubility experiments revealed Qu’s high solubility in ethanol, methanol, and dimethyl formamide, which can enhance its bioavailability and formulation versatility [27]. The measurement of pH at 2.2 indicated the drug’s compatibility with physiological conditions, with potential implications for gastrointestinal absorption.

FTIR spectroscopy confirmed the drug’s characteristic peaks, validating its authenticity and purity [39]. The FTIR analysis of reactants in Qu-AgNPs’ synthesis revealed key functional groups. The observed O-H stretching at 3732.67 cm^−1^ and peaks at 1519.12 cm^−1^ and 1659.48 cm^−1^ for (C=O stretch), 1453.78 cm^−1^ (C=C stretch), and 1012.67 cm^−1^ represent the stretching vibrations of the C-O bonds in Qu. These findings support Qu’s role in reducing and stabilizing Qu-AgNPs.

Furthermore, the development of a standard graph for Qu’s absorption at 420 nm in ethanol facilitated quantitative analysis, a valuable tool for determining drug concentrations in solutions. The formulation of Qu nanoparticles demonstrated a high drug entrapment efficiency, ideal particle characteristics for enhanced drug solubility, and stability, as revealed by TEM, zeta potential calculation, and particle size measurement [40,41,42,43]. TEM analysis, conducted at a magnification of 13.0 × 4000 with a scale bar set at 200 nm, offered a detailed view of the selected formulation. The structural characteristic is highly advantageous for pharmaceutical applications as it can significantly improve drug solubility and enhance bioavailability. The uniformity and stability of these vesicles indicate their potential in drug delivery systems, where solubility and bioavailability enhancement are crucial for effective therapeutic outcomes. This TEM analysis underscores the promising nature of this formulation for pharmaceutical and biomedical applications.

The zeta potential obtained demonstrates an excellent nanoparticle stability, a critical factor in preventing aggregation and extending shelf life. The finding underscores the formulation’s ability to maintain particle dispersion, which is vital for the practical application and storage of these nanoparticles. The Z-average particle size is well-aligned with the typical range observed in drug delivery nanoparticles. This size falls within a favorable range for potential therapeutic applications, as it offers advantages such as enhanced drug encapsulation and cellular uptake. The relatively uniform particle size further suggests consistency in the formulation, which is crucial for drug delivery systems. Overall, these findings indicate the suitability of these nanoparticles for therapeutic applications, particularly in drug delivery.

The X-ray diffraction pattern suggested alterations in Qu’s physicochemical properties, potentially improving its dissolution rate and bioavailability [44]. The in vitro release kinetics indicated controlled and sustained drug release, promising enhanced therapeutic efficacy and reduced side effects [45].

The assessment of antibacterial activity against *E. coli* highlighted the potential of Qu in different solvents [42]. Sample 2, containing methanol and Qu, exhibited a stronger antibacterial effect, possibly due to the enhanced solubility and bioavailability of Qu in methanol. Sample 1, with Qu in water, also displayed antibacterial activity, underscoring the inherent antimicrobial properties of quercetin even in an aqueous medium. These results suggest the versatility of Qu as an antimicrobial agent with potential applications in various formulations, such as food preservation and pharmaceuticals [30,36,46].

In conclusion, the comprehensive analysis of AgNPs loaded with Qu in this study provides valuable insights into their pharmaceutical potential. The drug’s physicochemical properties, solubility characteristics, nanoparticle formulation, and antimicrobial activity open avenues for further research and development in drug delivery systems and antimicrobial agents. These findings underscore the importance of solvent choice in optimizing the therapeutic properties of AgNPs loaded with Qu. Future investigations should explore the underlying mechanisms of action and conduct detailed studies to unlock the full therapeutic potential of Qu in various formulations.

## 5. Conclusions

In conclusion, this study rigorously examined various aspects of Qu and its silver nanoparticles’ formulation. The drug’s authenticity was confirmed through organoleptic characteristics, melting temperature, and DSC analysis. The pH assessment indicated its compatibility with physiological conditions. FTIR spectroscopy validated the chemical composition and provided insights into the role of Qu in synthesizing Qu-AgNPs. Spectrophotometry allowed for the development of a calibration curve for accurate drug concentration determination. The characterization of Qu-AgNPs revealed their stability, size, and potential for drug delivery. Moreover, the study assessed the in vitro release kinetics and demonstrated promising antibacterial activity against *E. coli*. This research paves the way for utilizing Qu-AgNPs in pharmaceutical applications, offering controlled drug release and antibacterial potential.

## Data Availability

The data presented in this study are available in this article.
